# Left atrial appendage thrombus formation, potential of resolution and association with prognosis in a large real-world cohort

**DOI:** 10.1038/s41598-023-27622-3

**Published:** 2023-01-17

**Authors:** Martina Hautmann, Michael Zacher, Sophia Fuchs, Christian Muñoz Pérez, Akram Ahmidou, Sebastian Kerber, Sebastian Barth

**Affiliations:** 1 Department of Cardiology, Rhön-Klinikum Campus Bad Neustadt, Von-Guttenberg-Str. 11, 97616 Bad Neustadt a.d.Saale, Germany; 2 Department of Medical Documentation, Rhön-Klinikum Campus Bad Neustadt, Bad Neustadt a.d.Saale, Germany

**Keywords:** Cardiology, Medical research

## Abstract

Comprehensive data on factors influencing left atrial appendage (LAA) thrombus formation, resolution and impact on survival are limited. In this single-center, retrospective study 7759 (2010–2015) patients with symptomatic ongoing atrial fibrillation (AF) on admission were screened for LAA thrombi. 450 patients had LAA thrombi. 481 patients without LAA thrombi were randomly selected as controls. We assessed clinical, echocardiographic, laboratory parameters and long-term survival of both groups. Patients with LAA thrombi compared to controls were older, had more strokes, higher CHA_2_DS_2_ -VASc scores, worse renal function, less controlled diabetes, advanced heart failure, lower LAA emptying velocities, higher levels of cardiac and inflammatory markers (all *p* < 0.001). 56.3% of followed-up patients (304) dissolved their LAA thrombi. Chances of thrombus resolution increased with rising LAA flow velocities (OR 1.061, *p* = 0.022), whereas advanced age (OR 0.950, *p* < 0.001) and presence of permanent AF (OR 0.354, *p* < 0.001) decreased chances of thrombus resolution. Presence of LAA thrombi was associated with a markedly reduced 10-year survival probability (31% versus 69%). LAA thrombus formation is promoted by advanced structural heart disease, inflammation, diabetes and impaired renal function. Younger age, non-permanent AF and higher LAA flow velocities were predictors of thrombus resolution. Thrombus formation was associated with poor prognosis.

## Introduction

The leading causes of morbidity and mortality in AF are thromboembolic events and heart failure^1^. The LAA is the main source of thromboembolism. Cresti et al. showed that only 0.07% of atrial clots are outside the LAA in non-valvular AF^2^. A number of studies with a small number of patients identified factors that were associated with LAA thrombus formation including type of AF, low emptying velocities assessed by Doppler echocardiography, non- chicken wing morphology, renal dysfunction, D-dimer levels, reduced left ventricular systolic function (LV EF) and enlarged left atrial size^3–7^. LAA thrombus formation does not only prevent restoring sinus rhythm (SR), but also interferes with interventional procedures like LAA closure, left atrium and ventricular radiofrequency ablations, interventional therapy of mitral and tricuspid valve regurgitation and closure of ASD or PFOs with occluders. As LAA thrombi occur more frequently in patients with advanced heart failure, their presence excludes these vulnerable patients from procedures that have been shown to improve symptoms and prognosis such as rhythm control or interventional valve repair^8,9^. Therefore, it is of great interest, not only to identify treatable causes that promote LAA thrombus formation, but also to investigate to what extent thrombus formation is reversible. There are few studies available, that address the latter issue. Also, little is known whether LAA thrombus formation is a surrogate parameter for worse prognosis.

## Methods

### Patient cohort

Between January 2010 and December 2015, 7759 consecutive patients diagnosed with symptomatic AF were admitted to our heart center. Definition of the different types of AF (paroxysmal, persistent, permanent) followed the guidelines for treatment of AF 2012^10^. All patients received a transoesophageal echocardiography (TOE) to rule out thrombi. Only patients with ongoing AF at presentation were included. 450 patients with LAA thrombi were identified. No thrombi outside the LAA were reported. 481 (out of 2191) patients without LAA thrombi were randomly selected as controls. Out of the initial 450 patients with thrombi, 304 patients were available for follow-up. Presence of AF was documented by repeated electrocardiograms (ECG) throughout the observation period.

The primary therapeutic goal was to restore sinus rhythm. When no thrombus was present, sinus rhythm was restored with antiarrhythmic drugs (amiodarone, class I antiarrhythmics), electric cardioversion (e CV) and/or pulmonary vein isolation (PVI). There was no follow-up in patients without LAA thrombi and restored sinus rhythm. When a LAA thrombus was detected (“first hospital visit”, “first TOE”), a revisit with another TOE examination was scheduled 3–4 month later. In case of LAA thrombus persistence at the first revisit, another appointment was arranged in 3–4 month. All revisits included TOE examination. This was repeated up to four times. The therapeutic goal for patients with LAA thrombi on vitamin K antagonists was increased to an international normalized ratio (INR) of 2.5–3.5 to promote thrombus resolution. New oral anticoagulants (NOAKs) were given at the highest recommended dosages, if possible. Treatment included heart rate lowering medication, antihypertensive drugs, state- of- the- art heart failure medication and medication to treat cardiovascular risk factors. When LAA thrombi did not resolve or patients became asymptomatic under therapy and AF was accepted as permanent rhythm, AF was considered to be permanent. AF was also considered to be permanent, when repeated e CVs and PVI including pretreatment with amiodarone did not restore sinus rhythm. TOE reevaluation ended (“last hospital visit” or “last TOE”) either when LAA thrombus had resolved and procedures to restore sinus rhythm were safe to perform or when AF was declared as “permanent”. Patients with acute coronary syndromes and infections were excluded in this study.

Written informed consent was obtained from all patients for invasive procedures. Informed consent of patients was obtained to be contacted by phone. The study was approved by the ethics committee of the Philipps University of Marburg, Department of Medicine. All methods were performed in accordance with the relevant guidelines and regulations as outlined in the Declaration of Helsinki.

### Ultrasound examination

All patients underwent transthoracic echocardiography (TTE) and TOE exam within 24 h of admission. LAA sludge, defined as a static gelatinous echo-density, present throughout the cardiac cycle and absence of color flow within the LAA was categorized as LAA thrombus, as well as formed echo-dense masses. The LAA peak emptying velocities were obtained by pulsed- wave Doppler placed within the first third of the LAA orifice and averaged over a minimum of 5 consecutive cardiac cycles. E/eˊ ratios were calculated using the septal velocities for eˊ^11^. Determination of left ventricular (LV) function and chamber dimensions followed recommendations of the American Society of Echocardiography^12^. Valvular heart disease was considered when severity was at least moderate according to guidelines^13,14^.

### Determination of LAA morphology

LAA morphology was determined by computed tomography (CT) analysis. CT scans performed to assess pulmonary vein anatomy or for other reasons were used for analysis. Four different shapes were used to categorize LAA morphology: Cactus, chicken wing, windsock and cauliflower as described previously^5^.

### Measurement of left ventricular end diastolic pressure (LVEDP)

Heart catheterization was performed only in patients with a history of typical chest pains during exercise or at rest, signs of ischemia in ECG and/or dynamic changes in cardiac marker levels. In cases, when a ventriculography was performed, LVEDP was measured invasively with a pigtail catheter placed in the left ventricle.

### Statistical analysis

All quantitative variables were expressed as mean ± standard deviation (SD) and compared using Student’s unpaired or paired t-test. Qualitative data (nominal or ordinal scale) are reported as absolute numbers or percentages and were compared using the chi-square test.

All tests were two-tailed, and *p* values < 0.05 were considered to indicate statistical significance.

A multivariable logistic regression model was used in patients with LAA thrombi to determine independent variables that could predict probability of thrombus resolution. Only parameters that were available in over 90% in all patients with LAA thrombi were used. Risk was expressed as odds ratios (ORs) with 95% confidence intervals (CI)s. Goodness of the multivariable models was confirmed using the Hosmer–Lemeshow test. The mortality rates were analyzed using the Kaplan–Meier method.

All data analyses were performed using IBM SPSS Statistics for Windows (v. 27.0; IBM Corporation,Armonk, NY, USA).

## Results

### Prevalence of LAA thrombus in study population

**Figure 1:**
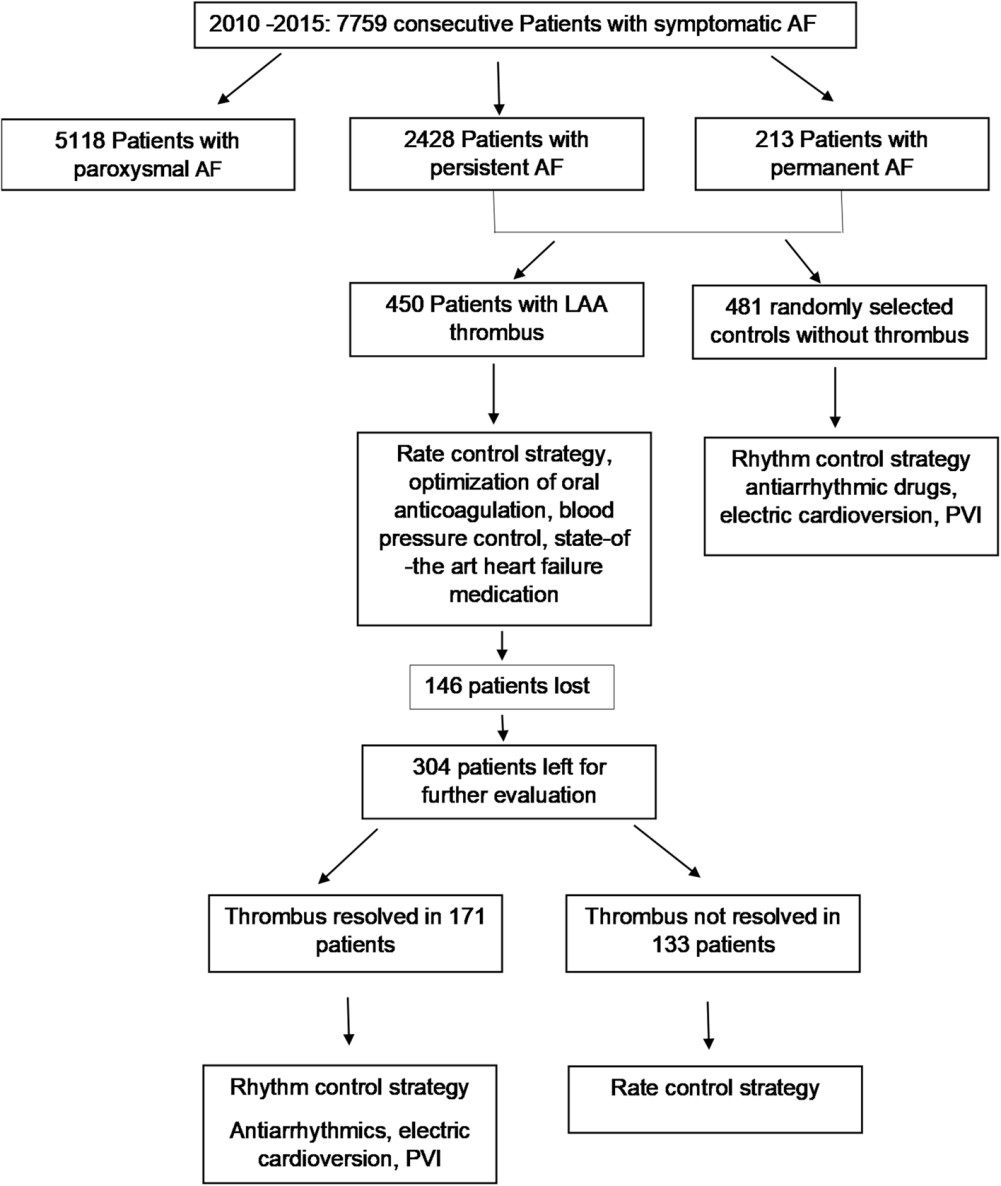
Enrollment and follow-up of study population.

Out of 7759 consecutive patients with symptomatic AF, 5118 patients suffered from paroxysmal AF (65.96%), 2428 patients from persistent AF (31.3%) and 213 from permanent AF (2.74%) on admission (Fig. 1). TOE examination identified a total of 450 (5.8%) LAA thrombi in patients with ongoing AF on admission. In patients with paroxysmal AF 0.2% LAA thrombi were found. Of the initial 450 patients with LAA thrombi, follow-up in 146 patients was not possible. Of the remaining 304 patients, 171 (56.3%) resolved their thrombi and received treatment to restore sinus rhythm. In most cases, thrombus resolution was achieved after 1–3 revisits. 133 (43.7%) patients did not dissolve their LAA thrombi and received medication to control heart rate.

### Comparison of groups with and without LAA thrombus

Table 1 shows a comparison of clinical, echocardiographic and laboratory parameters between both groups. Patients with LAA thrombi were significantly older (72.5 ± 8.8 vs. 67.8 ± 10.2 years, *p*** < **0.001), had more strokes (19.3% vs. 11.2%, *p*** < **0.001), higher rates of structural heart disease like dilated cardiomyopathy (DCM) and valvular heart disease such as mitral and aortic valve stenosis (all *p*** < **0.001). In patients with LAA thrombi CHA_2_DS_2_ -VASc scores ≥ 5 were present more than twice as often as compared to patients without thrombi (45.5% vs. 20.3%, *p*** < **0.001). The percentage of patients with combined coronary artery disease and peripheral artery disease was twice as high in the group with thrombus suggesting advanced atherosclerosis (21% vs. 9.6%, *p* < 0.001).

**Table 1:** Baseline characteristics of patients without and with LAA thrombi. Data are displayed as n (%) or mean ± SD. Actual numbers of patients differing from total number of study group are indicated separately for each parameter.

	Without LAA thrombus	With LAA thrombus	*p* value
	total *n* = 481	total *n* = 450	
Age (years)	67.8 ± 10.2	72.5 ± 8.8	** < 0.001**
Male	293 (60.9%)	292 (64.9%)	0.21
Body mass index (BMI) (kg/m^2^)	30.9 ± 6.0	30.2 ± 5.9	0.07
**Cerebral thrombotic events**			
Transient ischemic attack (TIA)	14 (2.9%)	20 (4.5%)	0.21
Stroke	54 (11.2%)	87 (19.3%)	** < 0.001**
		*n* = 432	**0.005**
Hypertension	442 (91.9%)	416 (96.3%)	
Diabetes mellitus Type II	110 (22.9%)	159 (35.3%)	** < 0.001**
**Atherosclerotic vessel disease**			
	*n* = 394	*n* = 374	** < 0.001**
Peripheral artery disease (PAD)	46 (11.7%)	90 (24.1%)	
	*n* = 459	*n* = 442	** < 0.001**
Coronary artery disease (CAD)	166 (36.2%)	237 (53.6%)	
One vessel	− 70 (42.4%)	− 93 (38.8%)	
Two vessels	− 42 (25.5%)	− 57 (23.8%)	
Three vessels	− 53 (32.1%)	− 84 (35.0%)	
	*n* = 387	*n* = 353	** < 0.001**
CAD + PAD	37 (9.6%)	74 (21.0%)	
**Structural heart disease**			
Dilated cardiomyopathy (DCM)	40 (8.3%)	85 (18.9%)	** < 0.001**
Hypertrophic (obstructive) cardiomyopathy H(O)CM	7 (1.4%)	10 (2.2%)	0.055
**Valvular heart disease (moderate and severe)**			
Mitral valve stenosis	*n* = 473		
	9 (1.9%)	30 (6.7%)	** < 0.001**
Mitral valve regurgitation	*n* = 467	*n* = 441	
	72 (15.4%)	89 (20.2%)	0.06
Prior mitral valve surgery and interventional procedures		*n* = 438	
	33 (6.8%)	47 (10.7)	0.17
Aortic valve stenosis	19 (4.0%)	55 (12.2%)	** < 0.001**
Prior aortic valve surgery/ interventional procedures	23 (4.7%)	32 (7.7%)	** < 0.001**
Aortic valve regurgitation		*n* = 428	
	17 (3.5%)	27 (6.3%)	0.052
Tricuspid valve regurgitation	*n* = 467		
	47 (10.1%)	121 (26.9%)	** < 0.001**
Prior reconstruction	*n* = 467		
	2 (0.4%)	12 (2.7%)	** < 0.001**
**Echocardiographic characteristics**			
Ejection fraction %	54.7 ± 12.9	42.6 ± 16.4	** < 0.001**
Septum diameter (mm)			
	*n* = 447	*n* = 431	** < 0.001**
≤ 13 mm	374 (83.6%)	320 (74.2%)	
≥ 14 mm	73 (16.3%)	111 (25.7%)	
Left atrium (LA) diameter parasternal long axis (mm)	*n* = 143	*n* = 138	**0.003**
	47.7 ± 7.2	50.2 ± 6.6	
LA area 4 chamber view (cm^2^)	*n* = 388	*n* = 394	
	27.4 ± 6.2	31.4 ± 7.1	** < 0.001**
Right atrium (RA) area 4 chamber view (cm^2^)	*n* = 293	*n* = 367	
	24.09 ± 5.1	27.12 ± 6.7	** < 0.001**
LAA flow velocity first TEE (cm/s)	*n* = 437	*n* = 421	
	44.0 ± 16.2	19.6 ± 5.6	** < 0.001**
E/e´	*n* = 215	*n* = 213	
	14.1 ± 4.5	19.1 ± 6.9	** < 0.001**
LVEDP (mmHg)	*n* = 191	*n* = 214	
	16.2 ± 5.3	17.9 ± 5.3	**0.002**
CHA_2_DS_2_- VASc score	*n* = 477	*n* = 448	** < 0.001**
≤ 4	380 (79.6%)	244 (54.5%)	
≥ 5	97 (20.3%)	204 (45.5%)	
**Blood chemistry characteristics**			
INR first admission	*n* = 236	*n* = 250	
	2.6 ± 0.76	2.38 ± 0.87	** < 0.001**
high-sensitivity (hs) Troponin T ng/ml (< 0.014 ng/ml)	*n* = 204	*n* = 225	
	0.023 ± 0.035	0.042 ± 0.049	** < 0.001**
NT-proBNP pg/ml (< 300 pg/ml)	*n* = 261	*n* = 325	
	2433.0 ± 2864.9	5687.9 ± 8596.9	** < 0.001**
D-Dimer mg/l (< 0.23 mg/l)	*n* = 129	*n* = 132	
	0.19 ± 0.2	0.31 ± 0.3	**0.002**
C-reactive protein (CRP) mg/dl (< 0.5 mg/dl)	*n* = 471	*n* = 444	
	0.37 ± 0.48	0.98 ± 2.01	** < 0.001**
Fibrinogen mg/dl (184–480 mg/dl)	*n* = 427	*n* = 381	
	378.1 ± 67.5	425.6 ± 76.3	** < 0.001**
HbA_1c_ mmol/mol (< 42 mmol/mol)	*n* = 211	*n* = 209	
	43.1 ± 10.5	47.2 ± 11.3	** < 0.001**
Glomerular filtration rate (GFR) ml/min/1.73m^2^		*n* = 448	
	73.8 ± 22.9	59.4 ± 22.5	** < 0.001**
**Oral anticoagulation on admission**			
None	72 (15.0%)	120 (26.7%)	
Vitamin K antagonist	253 (52.6%)	266 (59.1%)	
Factor Xa inhibitor	97 (20.2%)	38 (8.4%)	
Thrombin inhibitor	13 (2.7%)	10 (2.2%)	
Heparin low molecular weight	42 (8.7%)	12 (2.7%)	
**LAA morphology (CT)**	*n* = 251	*n* = 163	0.107
Cactus	71 (28.3%)	64 (39.3%)	
Chicken wing	114 (45.4%)	61 (37.4%)	
Windsock	50 (19.9%)	26 (16.0%)	
Califlower	16 (6.4%)	12 (7.4%)	

Significant values are in bold.

Echocardiographic parameters revealed a significantly lower left ventricular ejection fraction (LVEF), higher septal diameters, larger left and right atrial sizes and more prominent diastolic dysfunction (all *p*** < **0.001) in the group with LAA thrombus as compared to the group without. Consistent with the latter, the group with LAA thrombus had higher left ventricular end-diastolic pressures. In particular, LAA emptying velocities in the group with LAA thrombus were less than half of those observed in the group without thrombus (44.0 ± 16.2 vs. 19.6 cm/s ± 5.6; *p* < 0.001).

Blood work showed that patients with thrombi had significantly higher levels of troponin T, fibrinogen, C-reactive protein (CRP) and D-Dimers (all *p* < 0.001). Diabetes was less well controlled in the group with LAA thrombus and the degree of renal dysfunction was more advanced (all *p* < 0.001).

In the group with LAA thrombus 26.7% had no oral anticoagulants at first contact, whereas in the group without thrombus 15% had none. In the group with LAA thrombus, there were more patients who presented at first contact with signs of heart failure like dyspnea, pleural effusions and edema, unaware of tachyarrhythmia as underlying cause and therefore without oral anticoagulation. More patients in the group without thrombus were treated with NOACs (22. 9% vs. 10.6%). INR values of patients treated with vitamin K antagonists were within therapeutic range in both groups.

Since LAA morphology was shown to be a risk factor for stroke and formation of LAA thrombus^5^, we also investigated whether LAA morphology contributed to LAA thrombus formation. Non—chicken wing morphology was associated with a higher prevalence of stroke^5^. Although, there was a tendency for a higher frequency of non- chicken wing morphology in our study in the group with LAA thrombus (62.7% versus 54.6% in group without thrombus), the difference did not reach statistical significance (*p* = 0.107).

### Correlation of biomarkers, markers of inflammation and renal function with LAA thrombus formation

**Figure 2:**
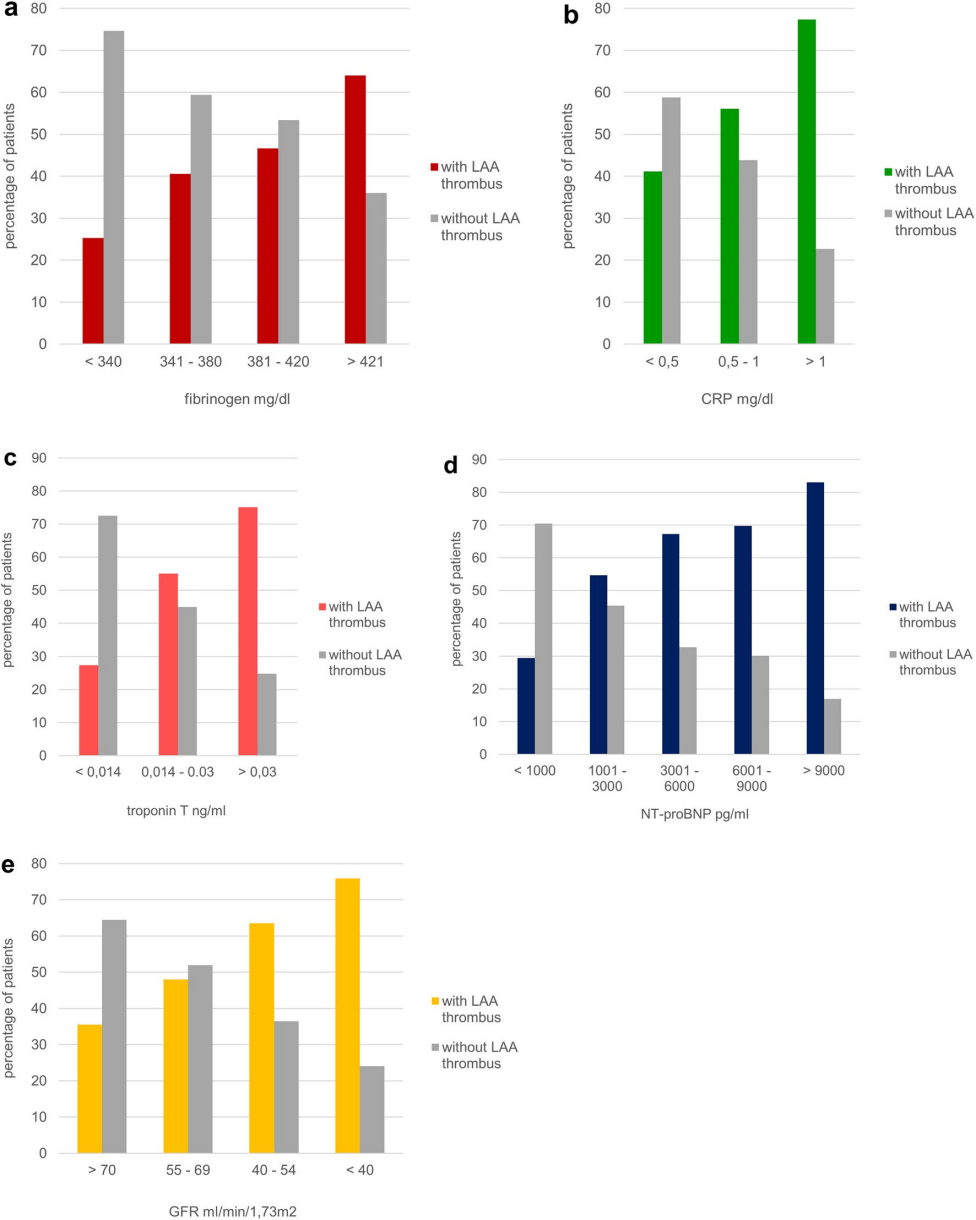
Correlation between different fibrinogen (**a**), C- reactive protein (CRP) (**b**), troponin T (**c**) and NT-proBNP (**d**) concentrations in serum and percentage of patients with and without LAA thrombus**.** Correlation between different Glomerular filtration rate (GFR) levels and percentage of patients with and without LAA thrombus (**e**).

Figure 2 a–d illustrates different concentration ranges of fibrinogen, CRP, troponin T and NT-proBNP in relation to percentages of patients with or without LAA thrombi. The percentage of patients with LAA thrombi rose with each level of these parameters (for further details see Fig. 2 a–d). More than half (64%) of patients with LAA thrombi had fibrinogen concentrations exceeding 421 mg/dl and 77.3% had CRP concentrations > 1 mg/dl (Fig. 2 a and b). 83% of patients with LAA thrombi showed NT-proBNP levels > 9000 pg/ml and 73.8% troponin T concentrations > 0.030 ng/ml (Fig. 2 c and d). An inverse relationship was found between glomerular filtration rate (GFR) and patients with LAA thrombi. The lower the GFR, the higher the percentage of patients with LAA thrombi was. In 75.9% of patients with LAA thrombi, GFR was < 40 ml/min/1.73m^2^ (Fig. 2e).

### Comparison of groups of patients that resolved versus did not resolve LAA thrombi

Baseline characteristics are shown in Table 2. Patients with persistent LAA thrombi were significantly older (*p* < 0.001), had more strokes (*p* = 0.012) and had significantly higher CHA_2_DS_2_-VASc scores (*p* = 0.002). In the group with persistent LAA thrombus, prevalence of tricuspid valve regurgitation including reconstruction was higher (*p* = 0.012), and the sizes of the right atrium were larger (*p* < 0.01).

**Table 2:** Baseline characteristics of patients with dissolved and persistent LAA thrombi. Data are displayed as n (%) or mean ± SD. Actual numbers of patients differing from total number of study group are indicated separately for each parameter.

	LAA thrombus dissolved	LAA thrombus persistent	*p-*value
	Total *n* = 171	Total *n* = 133	
Age (years)	69.9 ± 8.7	73.8 ± 8.2	** < 0.001**
Male	117 (68.4%)	90 (67.7%)	0.12
BMI (kg/m^2^)	*n* = 163	*n* = 126	
	31.1 ± 6.9	30.0 ± 4.9	0.154
**Cerebral thrombotic events**			
TIA	5 (2.9%)	5 (3.8%)	0.218
Stroke	26 (15.2%)	37 (27.8%)	**0.012**
Hypertension	*n* = 168	*n* = 130	
	160 (95.2%)	127 (97.7%)	0.538
Diabetes mellitus Type II	48 (28.1%)	50 (37.6%)	**0.032**
**Atherosclerotic vessel disease**			
PAD	*n* = 153	*n* = 112	
	30 (19.6%)	30 (26.8%)	0.24
CAD	*n* = 169		0.626
	77 (45.5%)	78 (58.6%)	
One vessel	34 (44.1%)	27 (34.6%)	
Two vessels	20 (26%)	17 (21.8%)	
Three vessels	23 (29.9%)	34 (43.6%)	
CAD + PAD	*n* = 145	*n* = 104	
	24 (16.6%)	24 (23.1%)	0.222
**Structural heart**			
DCM	40 (23.4%)	21 (15.8%)	0.16
HOCM/HCM	2 (1.2%)	3 (2.3%)	0.398
**Valvular heart disease (moderate and severe)**			
Mitral valve stenosis	5 (2.9%)	6 (4.4%)	** < 0.001**
Mitral valve regurgitation	i = 166	*n* = 130	
	28 (16.9%)	22 (16.9%)	0.05
Prior mitral valve surgery and interventional procedures	*n* = 167	*n* = 128	
	20 (11.9%)	12 (9.4%)	0.304
Aortic valve stenosis and prior aortic valve surgery/interventional procedures	13 (7.6%)	11 (8.3%)	**0.015**
	12 (7.0%)	10 (7.5%)	
Aortic valve regurgitation	*n* = 161	*n* = 127	
	4 (2.5%)	10 (7.9%)	**0.037**
Tricuspid valve Regurgitation and prior reconstruction	30 (17.5%)	41 (30.8%)	**0.012**
**Echocardiographic characteristics**			
Ejection fraction %	41.8 ± 17.0	44.1 ± 15.4	0.051
Septum diameter (mm)			
	*n* = 167	*n* = 128	0.536
≤ 13 mm	148 (88.6%)	103 (80.5%)	
≥ 14 mm	19 (11.4%)	25 (19.5%)	
LA diameter parasternal long axis (mm)	*n* = 55	*n* = 36	
	50.3 ± 8.2	51.1 ± 4.9	0.601
LA area 4 chamber view (cm^2^)	*n* = 158	*n* = 120	0.129
	30.5 ± 7.6	31.8 ± 6.9	
RA area 4 chamber view (cm^2^)	*n* = 142	*n* = 114	
	25.8 ± 5.5	27.6 ± 6.0	**0.010**
LAA emptying flow velocity (cm/s)		First TOE	0.836
		*n* = 129	
		18.7 ± 4.8	
		Last TOE	
		*n* = 117	
		18.1 ± 4.9	
LAA emptying flow velocity (cm/s)	First TOE		** < 0.001**
	*n* = 161		
	20.3 ± 5.3		
	Last TOE		
	*n* = 159		
	32.7 ± 11.9		
E/e´	*n* = 90	*n* = 72	
	18.2 ± 6.7	19.6 ± 6.0	0.177
LVEDP (invasive measurement. mmHg)	*n* = 84	*n* = 61	
	18.5 ± 5.3	17.9 ± 5.6	0.517
CHA_2_DS_2_- VASc score			**0.002**
≤ 4	113 (66.1%)	64 (48.1%)	
≥ 5	58 (33.9%)	69 (51.9%)	
**Blood chemistry characteristics**			
INR first admission	*n* = 122	*n* = 88	
	2.13 ± 0.63	2.34 ± 1.2	0.109
INR last admission	*n* = 115	*n* = 92	
	2.70 ± 0.66	2.80 ± 0.97	0.352
Duration of oral anticoagulation (between first and last admission in days)	*n* = 169	*n* = 124	
	145 ± 215	184 ± 222	0.139
hsTroponin ng/ml (< 0.014 ng/ml)	*n* = 87	*n* = 63	
	0.028 ± 0.02	0.046 ± 0.03	** < 0.001**
NT-proBNP pg/ml (< 300 pg/ml)	*n* = 130	*n* = 96	
	4284 ± 4880	5737 ± 9772	0.144
D- Dimer mg/l (< 0.23 mg/l)	*n* = 50	*n* = 37	
	0.25 ± 0.28	0.28 ± 0.25	0.609
C-reactive protein mg/dl (< 0.5 mg/dl)	*n* = 169		
	0.57 ± 0.74	0.74 ± 0.77	0.068
Fibrinogen mg/dl (184–480 mg/dl)	*n* = 150	*n* = 126	**0.015**
	412.3 ± 74.2	433.8 ± 71.1	
HbA_1c_ (mmol/mol) (< 42 mmol/mol)	*n* = 72	*n* = 56	**0.028**
	46.1 ± 11.3	50.58 ± 11.2	
GFR ml/min/1.73m^2^	64.5 ± 21.5	56.1 ± 20.85	**0.001**
**Oral anticoagulation at last admission**			
No effective oral anticoagulation	38 (22.2%)	16 (12%)	
Vitamin K antagonist	115 (67.3%)	92 (69.2%)	
Factor Xa inhibitor	14 (8.2%)	11 (8.2%)	
Thrombin inhibitor	2 (1.2%)	6 (4.5%)	
Heparin low molecular weight	2 (1.2%)	7 (5.3%)	
Heparin unfractionated	0 (0.0%)	1 (0.8%)	

Significant values are in bold.

**Figure 3:**
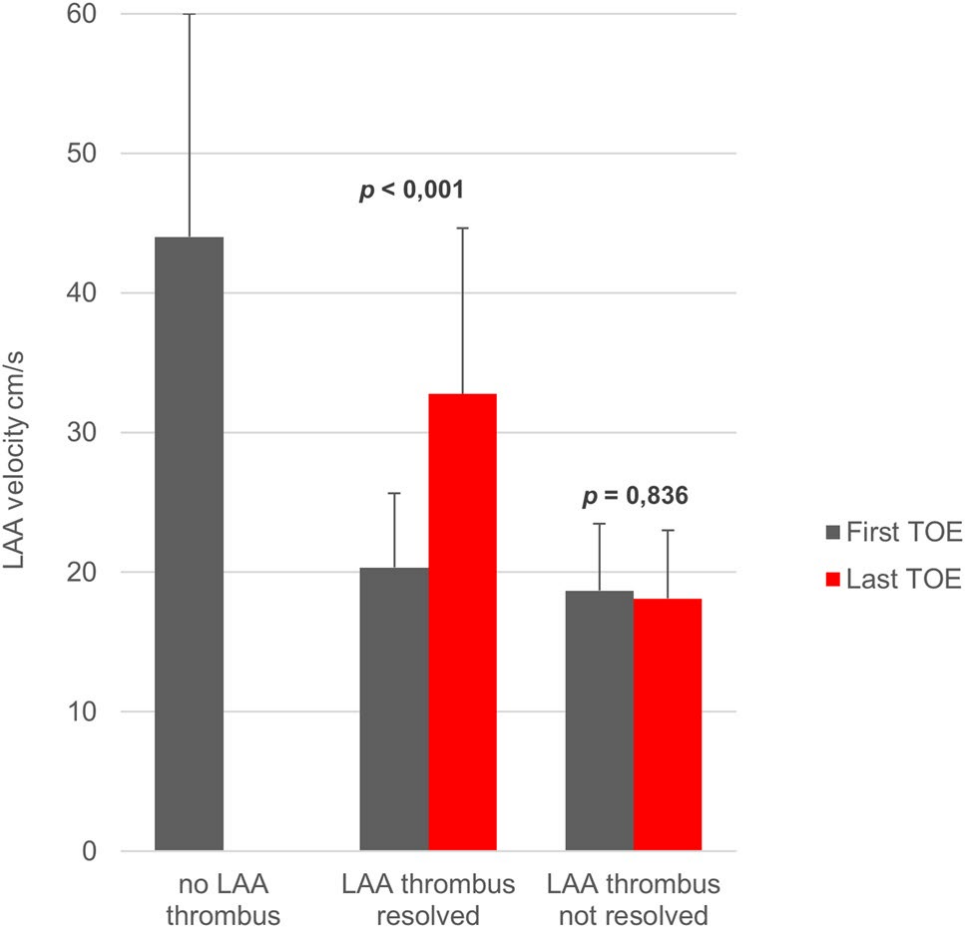
Comparison of LAA flow velocities. LAA flow velocities were measured in patients without LAA thrombi and in patients with LAA thrombi. LAA flow velocities of patients who dissolved their LAA thrombi and of patients that did not dissolve their LAA thrombi were measured at first contact (first TOE) and last contact, when LAA thrombi had either resolved or not resolved under intensified treatment (last TOE).

In the group that dissolved the LAA thrombus, LAA flow velocities measured in the last TOE were significantly higher compared to those in the first TOE (32.7 ± 11.9 vs 20.3 ± 5.3, *p* < 0.001), whereas they did not change in the group that did not dissolve LAA thrombus (18.1 ± 4.9 cm/s vs.18,7 ± 4,8 cm/s, *p* = 0.836, Fig. 3).

Blood chemistry revealed worse renal function, higher levels of troponin T and fibrinogen (*p* < 0.001) and a less well controlled diabetes mellitus (*p* = 0.028) in patients with persistent LAA thrombi. In both groups, INR values had increased during observation period. The percentage of patients without effective anticoagulation at the day of last admission was 12% in the group with persistent thrombus and 22.2% in the group with dissolved LAA thrombus. In these cases, patients were advised by their physicians to stop their oral anticoagulation 2–3 days before admission to minimize bleeding risk for expected invasive procedures. Duration of oral anticoagulation between first and last hospital visit did not statistically differ between patients who dissolved their LAA thrombi and those, who did not (145 ± 215 vs. 184 ± 222 days, *p* = 0.139).

### Comparison of success in restoring sinus rhythm in group without LAA thrombus and group with dissolved thrombus

Table 3 shows immediate success rates for restoring sinus rhythm when PVI and/or e CV were performed in different groups at the end of the last hospital stay. With PVI alone, sinus rhythm could be achieved in 95.6% of patients without LAA thrombi and in 66.6% of patients with dissolved LAA thrombi (*p* < 0.003). Similar results were observed, when electric cardioversion in corresponding groups was performed (93.9% versus 68.5%, *p* < 0.001). Success rates for sinus rhythm were higher, when a combined strategy (PVI and e CV) was applied (98.2% in the group without thrombus and 83.6% in the group with dissolved thrombus (*p* < 0.001).

**Table 3:** Comparison of success rates in restoring sinus rhythm with e CV and/or PVI in the group without LAA thrombus and group with dissolved LAA thrombus.

	without LAA thrombus	LAA thrombus dissolved	*p* value
Isolated pulmonary vein isolation (PVI)	*n* = 46	*n* = 21	***p*** **< 0.003**
Successfully restored SR	44 (95.6%)	14 (66.6%)	
Persistent atrial fibrillation	2 (4.4%)	6 (28.6%)	
Atypical atrial flutter	0 (0.0%)	1 (4.8%)	
Isolated electric cardioversion (e CV)	*n* = 132	*n* = 83	***p*** **< 0.001**
Successfully restored SR	124 (93.9%)	56 (68.5%)	
- Persistent atrial fibrillation	8 (6.1%)	26 (31.3%)	
Atypical atrial flutter	0 (0.0%)	1 (1.2%)	
Combination of pulmonary vein isolation and electric cardioversion	*n* = 224	*n* = 67	***p* < 0.001**
Successfully restored SR	220 (98.2%)	56 (83.6%)	
Persistent atrial fibrillation	4 (1.8%)	10 (14.9%)	
Atypical atrial flutter	0 (0.0%)	1 (1.5%)	

Significant values are in bold.

### Identification of independent variables influencing likelihood of LAA thrombus resolution

A logistic regression analysis was performed in patients with LAA thrombi to identify predictors of LAA thrombus resolution (Table 4). The independent variables age, type of AF (permanent or persistent) and LAA emptying flow velocities were found to be significant. Each additional year of life decreased the chance of dissolving LAA thrombus by 0.95-fold (*p* = 0.001). Patients with persistent AF had a 2.82 times greater chance of dissolving their LAA thrombus than patients with permanent AF (odds ratio = 0.354, *p* = 0.001). Each increase of velocity by 1 cm/s enhanced the chance of LAA thrombus dissolution by 1.061- fold (*p* = 0.022).

**Table 4:** Logistic regression analysis of predictors of LAA thrombus resolution.

	Regression coefficient	Standard error	Wald-test	df	*p*	OR	95% CI
Age per year	− 0.051	0.015	11.503	1	0.001	0.950	0.923–0.979
Type of AF	− 1.039	0.315	10.901	1	0.001	0.354	0.191–0.656
LAA emptying flow (per cm/s)	0.06	0.026	5.253	1	0.022	1.061	1.009–1.117
Constant	2.940	1.21	5.95	1	0.015	18.918	

### Association of LAA thrombus with long-term all-cause mortality

**Figure 4:**
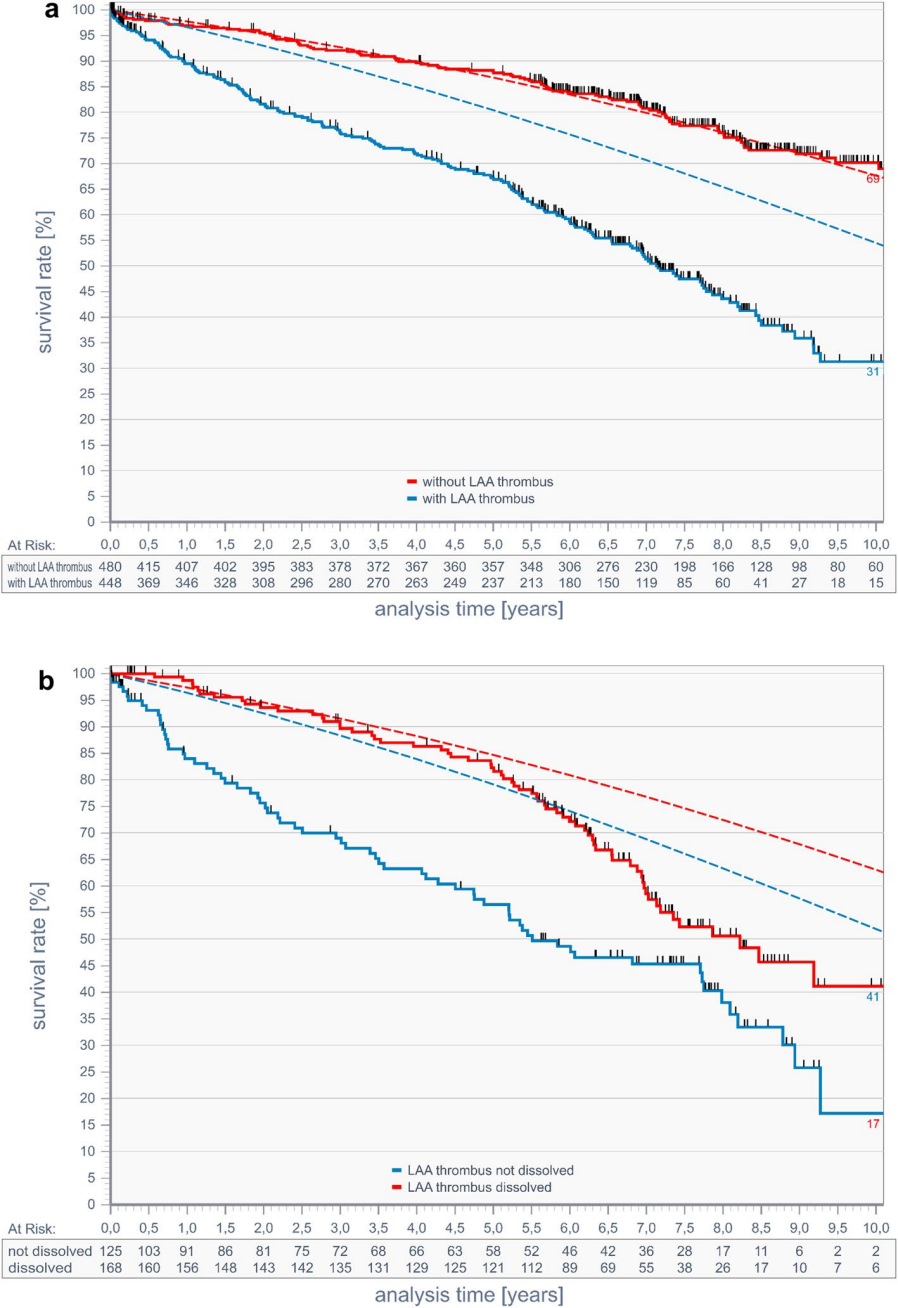
Comparison of all-cause mortality of patients without (red line) and with LAA thrombi (blue line). Dotted lines represent estimated all-cause mortality of an age-and sex-matched general population in Germany corresponding to each of the two groups (**a**). Comparison of all-cause mortality of patients who did not dissolve LAA thrombi (blue line) and of patients who dissolved LAA thrombi (red line). Dotted lines represent estimated all-cause mortality of an age-and sex-matched general population in Germany corresponding to each of the two groups (**b**).

The 10-year survival rate for patients without LAA thrombi was 69% and 31% in the group with LAA thrombi (Fig. 4a). Patients without LAA thrombi had the same all- cause mortality rate as an age-and sex-matched general population^47^, whereas all- cause mortality of patients with LAA thrombi was higher compared to an age-and sex-matched general population (31% vs 54%). Patients who dissolved their LAA thrombi had a better 10 year-survival compared to those, who did not (41% vs 17%, Fig. 4b). Survival rates of patients who dissolved LAA thrombi compared with the age- and sex-matched group showed reduced survival after 10 years (41% vs 63%) with curves diverging after 4.8 years. Survival rates of patients with persistent LAA thrombi were much worse than that of the age- and sex-matched group (17% vs 52%).

## Discussion

To our knowledge, we not only present the largest cohort of patients with LAA thrombi, but also provide a comprehensive assessment of a large number of clinical, echocardiographic and laboratory parameters, some of which previously shown in smaller studies (13–126 patients) to be involved in thrombus formation^3–7,15–22^. In addition, we identified factors associated with LAA thrombus resolution/persistence. A new finding was, that patients with LAA thrombi had a much worse long-term survival.

Thrombus formation occurred primarily in patients with persistent or permanent AF. Two studies^23,24^ showed that patients with persistent or permanent AF had a higher risk of stroke than patients with paroxysmal AF. Clinical features of patients with LAA thrombi in our study compared to those without were an advanced age, higher CHA_2_DS_2_-VASc scores, a higher number of previous strokes, more severe atherosclerosis and a higher incidence of structural heart disease including valvular heart disease. Palmer et al. has shown^25^ that LAA thrombus formation was present in one-third of patients with AF and severe aortic stenosis. In patients with severe mitral stenosis, incidence of LAA thrombus was higher than in patients with mitral regurgitation and controls^26^.

Interstitial fibrosis promoted by elevated left-sided filling pressures was shown to result in LA stiffness and impaired LA contractility^27^. As a result, pulmonary pressures increase and lead to right heart dilatation and tricuspid valve regurgitation^28^. In line with these pathophysiological events, we could show that patients with LAA thrombi had more advanced systolic and diastolic LV dysfunction, larger atrial sizes, very low LAA emptying velocities and significant tricuspid regurgitation. Presence of congestive heart failure and diastolic dysfunction were found to be independent predictors of LAA thrombus^7,18,20^.

We found that markers of cardiac strain and damage like NT-pro BNP and hs troponin T were markedly elevated in patients with LAA thrombi, an observation also reported by previous studies^15,16^. Moreover, we could demonstrate a stepwise increase of percentage of patients with LAA thrombi with increasing values of hs troponin T, NT-pro-BNP, fibrinogen and CRP (Fig. 2 a–d) suggesting that extent of cardiac strain and inflammation were associated with thrombus formation and thrombus persistence. Berg et al.^29^ showed that troponin T, NT-proBNP, age and history of stroke were the strongest predictors of stroke and systemic embolism. In addition, elevated hs troponin and BNP levels were not only found to be associated with low LAA flow velocities and incidence of LAA thrombus but also linked to worse prognosis in patients with AF^15,16,30^. Addition of a number of these factors to the CHA_2_DS_2_- VASc score could improve prediction of stroke and LAA thrombus formation^7,19^.

In our study, a poorly controlled diabetes and advanced renal dysfunction were associated with LAA thrombus persistence. Numerous studies indicated that both conditions were associated with increased inflammation, coagulation pathologies and atrial fibrosis^31,32^. Advanced kidney failure and dialysis are known risk factors for AF and thromboembolic events^33^. A reduced GFR was found to be an important predictor of LAA thrombus^3^.

So far, fibrinogen has not been shown to be associated with LAA thrombus formation before. In patients with advanced chronic kidney disease, fibrinogen levels were an independent predictor of mortality^34^. In diabetics, fibrinogen levels were elevated^35^. AF was found to create a thrombogenic milieu by multiple cascades in the LA including prothrombic endothelial changes^36,37^, platelet activation and thrombin generation. It is conceivable that systemic elevation of prothrombotic factors like fibrinogen and prothrombotic events in the LA induced by persistent AF may reinforce each other to promote thrombus formation.

Few studies have addressed the question to what extent LAA thrombi dissolve and which factors other than oral anticoagulants might be involved in this process. Consistent with other studies^21,38,39^, more than half of our patients with initial LAA thrombus dissolved their LAA thrombi. We showed for the first time that patients who resolved their thrombi significantly increased average LAA flow velocities, while still in AF. LAA flow velocities during AF were shown to be modulated by ventricular heart rate^40^. Longer cardiac cycles were associated with higher mean LAA velocities^40^. Furthermore, there is evidence that LA pressure is an important determinant of LAA flow. Treatment resulting in lower LA filling pressures was accompanied by improved LAA contractions^41^. Both observations encourage strict control of blood pressure and heart rate in patients with LAA thrombi. Although not yet investigated, resolution of the LAA thrombus itself might also contribute to improved LAA velocities, since its presence could have reduced available volume of the LAA and affected its mechanics. Higher LAA flow velocities during AF identified patients with a greater likelihood to remain in sinus rhythm one year after successful e CV^42^. Our results also showed that patients without LAA thrombi had much higher LAA flow velocities and a higher success rate in restoring sinus rhythm as compared to those who initially presented with a LAA thrombi and dissolved them.

A number of studies showed that flow velocities ≤ 20 cm/s were associated with LAA thrombus formation and a higher incidence of thromboembolic events^4,39,43^. Our patients with low LAA flow velocities that did not increase in response to treatment were also less likely to resolve their LAA thrombi.

At last, we show for the first time that 10-year survival was greatly reduced in patients with LAA thrombi compared to those with no thrombi. 10-year survival in patients with persistent LAA thrombi was also worse compared to those with dissolved thrombi. Differences in survival were still apparent when groups were compared to an age-and sex-matched general population. Although, patients with LAA thrombi have a higher burden of comorbidities that could account for observed differences, presence of LAA thrombi may further contribute to mortality by thromboembolic events. AF was shown to be associated with an increased risk of all-cause mortality, cardiovascular mortality, ischemic stroke and heart disease, sudden cardiac death, heart failure, chronic kidney disease and peripheral arterial disease^44^. LAA occlusion during cardiac surgery in patients with AF reduced the risk of ischemic stroke or systemic embolism^45^. Patients with LAA thrombi were shown to have a lower event-free survival from cardiovascular death than patients without thrombi^46^ suggesting thrombus formation as an additional factor for death. The Castle-AF study^8^ indicated that ablation of patients with AF and reduced LV EF improved LVEF and reduced all-cause mortality. This result suggested that not only the burden of diseases that promote AF was responsible for death but AF itself played an important role. More studies are needed to resolve this issue.

## Conclusions

LAA thrombus formation is a multifactorial process with numerous factors amplifying each other in a complex interplay resulting over time in irreversible structural changes of the atrial wall. This study extended current knowledge by following new findings:

Prevalence of LAA thrombi is associated with increasing concentrations of inflammatory parameters and markers of cardiac strain as well as declining renal function, pointing to a dynamic process of worsening organ functions. Fibrinogen has not shown before to be associated with LAA thrombus formation and persistence.

Comparison of patients who dissolved their LAA thrombi versus those who did not, was not done before. Factors identified to be associated with LAA thrombus persistence despite effective oral anticoagulation were badly controlled diabetes, advanced renal failure, high levels of troponin T and fibrinogen, as well as indicators of right ventricular dysfunction most likely being a result of long lasting left ventricular dysfunction and elevated pulmonary pressures.

Increases of LAA flow velocities in patients with LAA thrombi while still in AF predicted LAA thrombus resolution.

Short term success in restoring sinus rhythm in patients with dissolved thrombi was high, but significantly lower as compared to patients with no LAA thrombi.

Presence of a LAA thrombus was associated with a markedly increased all-cause mortality compared to patients without LAA thrombi even when compared to age- and sex-matched groups of a general population. Patient who dissolved their LAA thrombi (over 50%) had a better long-term prognosis than those with persistent LAA thrombi.

These results have important clinical implications. LAA thrombi, especially when persisting, are indicators for worse prognosis and associated with advanced renal and heart failure. Cardiovascular risk factors identified to play a role in LAA thrombus formation should be treated early and aggressively. State-of-the art device- and medical therapy needs to be applied to prevent heart failure and renal dysfunction from further deterioration. However, LAA thrombus is not an irreversible fate in a number of patients but should encourage physicians to intensify available treatment options.

### Study limitation

Data are based on a single center, retrospective study. A number of patients in the group diagnosed with a LAA thrombus did not come back to our institution after first contact and were lost for follow up. This might result in a sampling bias. In addition, there were more patients with LAA thrombi without oral anticoagulation, when admitted for the first time. This could also result in a bias comparing patients with and without LAA thrombi. Due to the retrospective nature of the study, a number of parameters investigated were not available for all patients preventing multi regression analysis with all of the parameters investigated*.* In addition, it was not possible to evaluate patient compliance with medication. However, the fact that INR values were within the therapeutic range and increased over the observation period may indirectly indicate compliance. There was no long- term follow up for rhythm control in patients of the different groups available.

## Data Availability

All datasets used in the current study are available from the corresponding author upon reasonable request.
